# Uterine leiomyoma causes an increase in systolic blood pressure: a two-sample Mendelian randomization study

**DOI:** 10.3389/fendo.2024.1373724

**Published:** 2024-05-10

**Authors:** Hui Xu, Yuxia Ma, Yi Long, Ren Liu, Ziyang Cheng, Xiuzhen Xie, Xingjun Han, Xuan Wang

**Affiliations:** ^1^ Obstetrics and Gynecology Department, The Second Affiliated Hospital of Shandong University of Traditional Chinese Medicine, Jinan, China; ^2^ The First Clinical College, Shandong University of Traditional Chinese Medicine, Jinan, China; ^3^ College of Acupuncture, Moxibustion and Tuina, Shandong University of Traditional Chinese Medicine, Jinan, China; ^4^ Shandong Provincial Traditional Chinese Medicine Data Center Management Office, The Second Affiliated Hospital of Shandong University of Traditional Chinese Medicine, Jinan, China; ^5^ Medical Affairs Office, The Fifth Affiliated Hospital Sun Yat-sen University, Zhuhai, China; ^6^ Disease Prevention Center, The Second Affiliated Hospital of Shandong University of Traditional Chinese Medicine, Jinan, China

**Keywords:** uterine leiomyoma, hypertension, hypertensive disorders of pregnancy, Mendelian randomization, causal relationship

## Abstract

**Objectives:**

Hypertension and hypertensive disorders of pregnancy (HDP) are common diseases in women at different stages, which affect women’s physical and mental health, and the impact of the latter on the offspring cannot not be ignored. Observational studies have investigated the correlation between uterine leiomyoma (UL) and the above conditions, but the relationship remains unclear. In this study, we employed two-sample Mendelian randomization (MR) analysis to assess the association between UL and hypertension, HDP, as well as blood pressure.

**Methods:**

We collected genetic association data of UL (35,474 cases), hypertension (129,909 cases), HDP (gestational hypertension with 8,502 cases, pre-eclampsia with 6,663 cases and eclampsia with 452cases), systolic blood pressure (SBP) and diastolic blood pressure (DBP) (both 757,601 participants) from published available genome-wide association studies (GWAS). The single nucleotide polymorphisms (SNPs) associated with UL phenotype were used as instrumental variables, and hypertension, three sub-types of HDP, SBP and DBP were used as outcomes. The inverse-variance weighted (IVW) method was employed as the primary method of causal inference. Heterogeneity was assessed using Cochran’s Q test, and sensitivity analyses were conducted using MR-Egger regression and MR pleiotropy residual sum and outlier (MR-PRESSO) tests to evaluate the pleiotropy of instrumental variables. PhenoScanner search was used to remove confounding SNP. Robustness and reliability of the results were assessed using methods such as the weighted median and weighted mode.

**Results:**

The IVW analysis revealed a positive correlation between genetically predicted UL and SBP [odds ratio (OR)= 1.67, 95% confidence interval (CI):1.24~2.25, *P* = 0.0007], and no statistical association was found between UL and hypertension, HDP, or DBP. The MR-Egger regression suggested that the above causal relationships were not affected by horizontal pleiotropy. The weighted median method and weighted model produced similar results to the IVW.

**Conclusion:**

Based on large-scale population GWAS data, our MR analysis suggested a causal relationship between UL and SBP. Therefore, women with UL, especially pregnant women, should pay attention to monitoring their blood pressure levels. For patients with hypertension who already have UL, interventions for UL may serve as potential therapeutic methods for managing blood pressure.

## Introduction

Hypertension is a common chronic disease. It is estimated that the number of global adults with hypertension is approaching 1 billion in 2000, and is projected to rise to 1.56 billion by 2025 (1), seriously affects people’s physical and mental health. Hypertensive disorder of pregnancy (HDP) has a high prevalence globally, affecting 3% to 5% of pregnant women worldwide. Nearly one-third of hospitalized women die from this disease, making it the leading cause of maternal mortality (2, 3). Chronic hypertension increases the incidence of pregnancy-related diseases and adverse pregnancy outcomes in women, such as hypertensive disorders of pregnancy, intrauterine growth restriction, preterm birth, and stillbirth (4, 5). Uterine leiomyoma (UL) is a common benign tumor in women of childbearing age, and it is associated with adverse pregnancy outcomes, including preterm delivery and placental abruption (6, 7). The prevalence of UL is difficult to accurately estimate, and some patients are severely underestimated due to clinical asymptomatic status. Statistically, the prevalence of UL ranges from 4.5% and 68.6% in different countries (8).

Over the years, many studies have investigated the relationship between UL and cardiovascular diseases (9–15), but the conclusions of the studies have not yet been unified. For example, Chen et al. (9) conducted a cross-sectional study and meta-analysis involving 8,401 patients, showing a significant correlation between UL and hypertension. In contrast, the Northern Finland Birth Cohort study of 3,635 participants analyzed the situation from birth to 46 years and found no association between UL and hypertension (14). Additionally, hormonal changes often occur during pregnancy, and estrogen and progesterone often play a key role in the development of UL (16). A study in the United States showed that UL is relatively common during pregnancy, with significant differences in prevalence among different ethnic groups (17). It is worth noting that a recent study found that UL in early pregnancy may increase the risk of HDP (18). Due to inconsistent results from observational studies and the limitations in causal inference that exist in traditional observational studies, such as confounding factors and reverse causality, it is necessary to further investigate the relationship between UL and hypertension and HDP.

Mendelian randomization (MR) is a research method in genetic epidemiology that evaluates the causal relationship between exposure and outcome with the help of genetic variants, such as single nucleotide polymorphisms (SNPs), which are used as instrumental variables. Since genes are randomly allocated at the time of conception and are not affected by external environment or social factors, MR can avoid the confounding effects and reverse causality that exist in the observational studies, making it a relatively precise epidemiological method (19, 20). Genome-wide association study (GWAS) is a method used to detect gene variations associated with complex human diseases or traits, and its objective is to reveal the impact of genetic variations on the risk of complex diseases, thereby providing new clues and strategies for prevention, diagnosis, and treatment (21). In this study, we explored the relationships between UL and hypertension, gestational hypertension, pre-eclampsia, and eclampsia using a two-sample MR study through GWAS summary data, and further investigated the association between UL and systolic blood pressure (SBP) and diastolic blood pressure (DBP).

## Materials and methods

### Data sources

The genetic instruments for UL were obtained from the published GWAS meta-analysis of UL in 2019 (22), which is publicly available. The GWAS meta-analysis included the Women’s Genome Health Study, Northern Finnish Birth Cohort, QIMR Berghofer Medical Research Institute, UK Biobank (UKBB), and the cohort studies from 23andMe. The study population included 35,474 cases and 267,505 controls, and all individuals were of European descent.

The genetic data for hypertension were obtained from the published GWAS of age-related diseases in 2021 (23), which extracted information from UKBB and contained 129,909 hypertension cases and 354,689 controls. There exist some overlap between the UL dataset and the hypertension dataset, accounting for up to 41.80% of the samples. However, the calculated probability of type I error due to this overlap was 0.05, with potential bias less than 1%, which can be considered negligible.

The genetic data for HDP were obtained from the FinnGen R9 biobank published in May 2023. We chose three sub-types: gestational hypertension, pre-eclampsia, eclampsia. The study population was all of European ancestry.

The genetic data for SBP and DBP were selected from the International Blood Pressure Consortium and UKBB with a total of 757,601 participants of European ancestry (24) (**Table 1**). Similarly, there was some overlap between the exposure dataset and the current dataset (26.74% of the samples), but the potential bias due to this overlap was calculated to be negligible, less than 1%.

**Table 1:** Sample sources in MR studies.

Trait	Sample size	Ancestry	Year	Website
Uterine leiomyoma	302,979	European	2019	https://www.nature.com/articles/s41467-019-12536-4
Hypertension	484,698	European	2021	https://www.ncbi.nlm.nih.gov/pmc/articles/PMC7610725/
Gestational [pregnancy-induced] hypertension	202,768	European	2023	https://storage.googleapis.com/finngen-public-data-r9/summary_stats/finngen_R9_O15_GESTAT_HYPERT.gz
Pre-eclampsia	200,929	European	2023	https://storage.googleapis.com/finngen-public-data-r9/summary_stats/finngen_R9_O15_PREECLAMPS.gz
Eclampsia	194,718	European	2023	https://storage.googleapis.com/finngen-public-data-r9/summary_stats/finngen_R9_O15_ECLAMPSIA.gz
Systolic blood pressure	757,601	European	2018	https://www.ncbi.nlm.nih.gov/pmc/articles/PMC6284793/
Diastolic blood pressure	757,601	European	2018	https://www.ncbi.nlm.nih.gov/pmc/articles/PMC6284793/

### Study design

**Figure 1:**
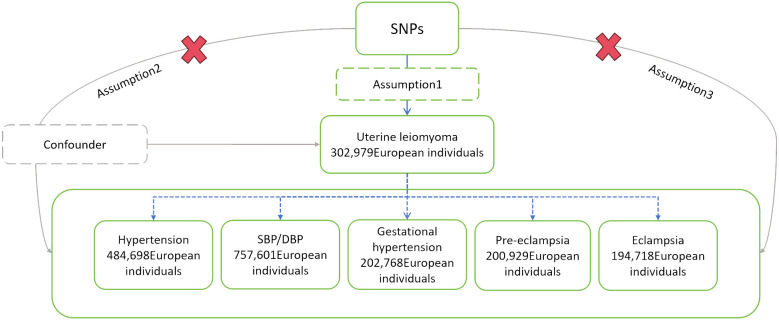
Schematic diagram of MR analysis. SNPs, single nucleotide polymorphisms; SBP, systolic blood pressure; DBP, diastolic blood pressure.

We tested the causal relationship between the exposure (UL) and each outcome (hypertension, the three sub-types of HDP, SBP, and DBP) using MR analysis. To make reasonable interpretations of MR analysis, three core assumptions must be satisfied (25). Firstly: Association - The genetic variations are strongly associated with exposure. Secondly: Independence - The genetic variations are independent of the confounders that affect the association between exposure and outcome. Thirdly: Exclusion - The genetic variations only affect the outcome through exposure (**Figure 1**).

### Selecting methods for instrumental variables

Genetic variations, SNPs, were used as instrumental variables, which were extracted from relevant GWAS (**Table 1**). The selected instrumental variables needed to satisfy the following conditions: (1) *P* < 5×10^−8^, SNPs were significantly correlated with UL at the genome-wide level; (2) *R* ^2^ = 0.001, genetic distance=10,000kb, remove linkage disequilibrium; (3) SNPs were not significantly associated with the outcome, setting *P* = 5×10^−5^; (4) SNPs did not have palindromic structures; (5) *F*>10, SNPs with an *F*-statistic less than 10 were excluded to avoid bias brought by weak instrumental variables. Weak instrumental variables are associated with exposure but have low explanatory capacity of exposure, providing little statistical power to test the hypothesis, which may lead to inaccurate estimation of causal effects and increased type I error probability (26). The strength of instrumental variables can be quantitatively evaluated using the *F*-statistic. The *F*-statistic for individual SNPs is calculated using the formula: *F* = *β* ^2^/*se* ^2^ *(* 27). According to traditional experience, *F* > 10 is less affected by the bias caused by weak instrumental variables (28).

### Statistical analysis

Using the Steiger Test to detect the presence of reverse causation for instrumental variables (29). The inverse-variance weighting (IVW) method is used as the main method for causal inference. This method assumes that all SNPs are valid and exist no pleiotropy, providing a well statistical power, but when SNPs have pleiotropy, the results may be biased (30, 31). Cochran' s Q test is used to assess heterogeneity among selected SNPs. The Q statistic is a weighted sum of squared deviations standardized by study variance. *P*<0.05 suggests heterogeneity, and a random effects model is used to assess causal associations, otherwise a fixed effects model is used (32). Because of the impact of pleiotropy on estimated association effects, MR-Egger regression is used to test for pleiotropy. If the intercept of the MR-Egger regression model is not zero (*P*<0.05), it indicates the presence of gene pleiotropy (33). The robustness of the results is analyzed using MR-Egger method, weighted median method and weighted mode. The MR-Egger method is mainly used for MR causal inference when there is potential pleiotropy (33). The weighted median method requires that at least 50% of the weights come from valid instrumental variables. It is the best choice when there is heterogeneity but no pleiotropy (34). The weighted model identifies multiple variables as valid instrumental variables to detect similar causal effects (35). When the main method (IVW) results are significant (*P*<0.05) and the other three methods agree with IVW, it can be considered that there is a causal relationship. Additionally, the MR pleiotropy residual sum and outlier (MR-PRESSO) method is used. If outliers are found, they are excluded and the causal association is re-estimated (36). To minimize the interference of horizontal pleiotropy on the results, each SNP was manually searched one by one in the human genotype-phenotype database PhenoScanner V2 (37) to identify and exclude risk factors shared with UL, hypertension and HDP, such as body mass index (BMI) (38, 39), waist circumference (40). Subsequently, SNPs with genome-wide significant associations (*P*<5×10^-8^) were selected, and causal inference was conducted anew.

Because this study has three subtypes of HDP, Bonferroni correction is used in order to reduce the probability of false-positive results. When *P*<0.017 (0.05/3), it indicates a significant causal relationship. The above methods are all performed using R 4.2.3. Statistical analysis was processed using the R Package Two Sample MR (v 0.5.8). The removal of outliers was conducted using the R Package MRPRESSO (v 1.0). And data visualization was conducted using the R Package forestploter (v 1.1.1) and CMplot(v 4.5.1).

## Result

### Incorporated instrumental variables

**Figure 2:**
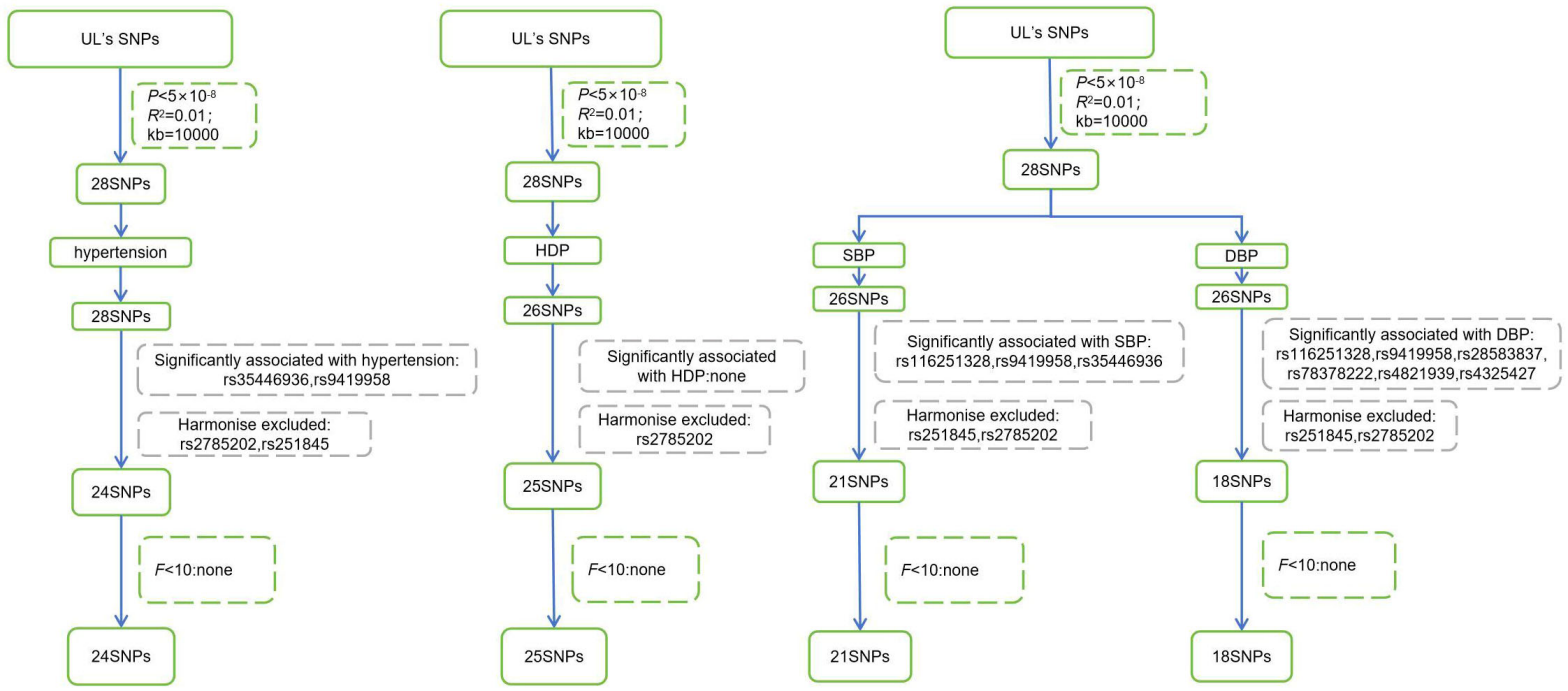
Steps for selecting instrumental variables. UL, Uterine leiomyoma; SNPs, single nucleotide polymorphisms; HDP, Hypertensive disorder of pregnancy; SBP, systolic blood pressure; DBP, diastolic blood pressure.

After selecting and harmonizing these instrumental variables (**Figure 2**), 24 SNPs were used for UL-hypertension MR-analysis, 25 SNPs were used for UL- three types of HDP MR-analysis, and UL-SBP and UL-DBP MR-analysis respectively included 21 SNPs and 18 SNPs (Those selected SNPs can be seen through **Supplementary Tables 1-4**). All instrumental variables passed the Steiger Test and no reverse causation was detected.

### Causal link between UL and outcomes

The Cochran' s Q test showed significant heterogeneity in the outcomes of hypertension, SBP, and DBP, so a random effects model was used for causal inference. No heterogeneity was found in the outcomes of gestational hypertension, pre-eclampsia, and eclampsia (*P*>0.05), so a fixed effects model was used. The *P*-values of the MR-Egger intercept test for each outcome did not show evidence of pleiotropy (**Table 2**).

**Table 2:** Results of heterogeneity and pleiotropy tests for instrumental variables.

Outcome	heterogeneity test		pleiotropy test
	*Q*	*P*	*P*
Hypertension	63.49	<0.01	0.69
Gestational hypertension	23.05	0.52	0.91
Pre-eclampsia	29.42	0.20	0.81
Eclampsia	14.47	0.94	0.28
Systolic blood pressure	78.10	<0.01	0.97
Diastolic blood pressure	41.72	<0.01	0.13

Genetic prediction suggested that UL may increase the risk of hypertension, gestational hypertension, pre-eclampsia, eclampsia, SBP and DBP, but none of these reached statistical significance. MR-Egger method, weighted median method and weighted model suggested that the causal association between UL and each outcome are consistent with IVW. It is worth mentioning that the MR-Egger analysis showed a significant association between UL and DBP [odds ratio, (OR)=1.62, 95% CI: 1.05~2.48, *P*=0.04]. However, the MR-Egger method is used for causal inference when there is potential pleiotropy, and the IVW method did not suggest a causal relationship, so it cannot be concluded that there is a causal association between UL and DBP (**Table 3**).

**Table 3:** UL’s causal inference results with each ending.

Outcome	Method	nSNP	*beta*	*se*	OR(95%CI)	*P*
Hypertension	Inverse variance weighted	24	<0.01	<0.01	1.01(1.00-1.02)	0.07
	MR Egger	24	0.01	<0.01	1.01(0.99-1.03)	0.26
	Weighted median	24	<0.01	<0.01	1.00(0.99-1.01)	0.51
	Weighted mode	24	<0.01	<0.01	1.00(0.99-1.01)	0.88
Gestational hypertension	Inverse variance weighted	25	0.03	0.05	1.03(0.94-1.13)	0.57
	MR Egger	25	0.02	0.10	1.02(0.84-1.23)	0.87
	Weighted median	25	0.03	0.07	1.03(0.90-1.17)	0.67
	Weighted mode	25	0.03	0.08	1.03(0.88-1.21)	0.69
Pre-eclampsia	Inverse variance weighted	25	0.04	0.06	1.04(0.93-1.17)	0.50
	MR Egger	25	0.01	0.13	1.01(0.79-1.29)	0.92
	Weighted median	25	0.02	0.08	1.02(0.88-1.19)	0.76
	Weighted mode	25	0.02	0.08	1.02(0.87-1.20)	0.81
Eclampsia	Inverse variance weighted	25	0.22	0.20	1.25(0.85-1.84)	0.26
	MR Egger	25	0.63	0.42	1.88(0.83-4.26)	0.15
	Weighted median	25	0.37	0.29	1.45(0.83-2.55)	0.19
	Weighted mode	25	0.37	0.38	1.44(0.69-3.02)	0.34
Systolic blood pressure	Inverse variance weighted	21	0.10	0.20	1.11(0.74-1.65)	0.62
	MR Egger	21	0.03	0.46	1.03(0.42-2.57)	0.94
	Weighted median	21	0.22	0.18	1.24(0.87-1.78)	0.24
	Weighted mode	21	0.65	0.51	1.92(0.70-5.26)	0.22
Diastolic blood pressure	Inverse variance weighted	18	0.17	0.09	1.18(0.98-1.42)	0.08
	MR Egger	18	0.48	0.22	1.62(1.05-2.48)	0.04
	Weighted median	18	0.10	0.09	1.11(0.93-1.32)	0.25
	Weighted mode	18	0.09	0.11	1.10(0.88-1.36)	0.42

nSNP, numbers of single nucleotide polymorphisms; OR, odds ratio; CI, confidence interval.

**Figure 3:**
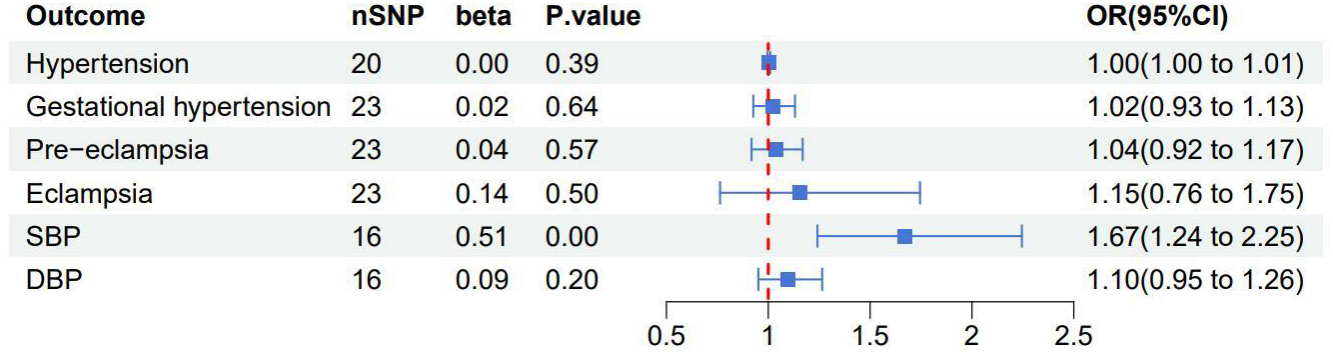
Forest plot of the results of the IVW method research between uterine leiomyoma and each outcome after excluding outliers and confounding SNPs associated with BMI related phenotypes. SBP, systolic blood pressure; DBP, diastolic blood pressure.

**Figure 4:**
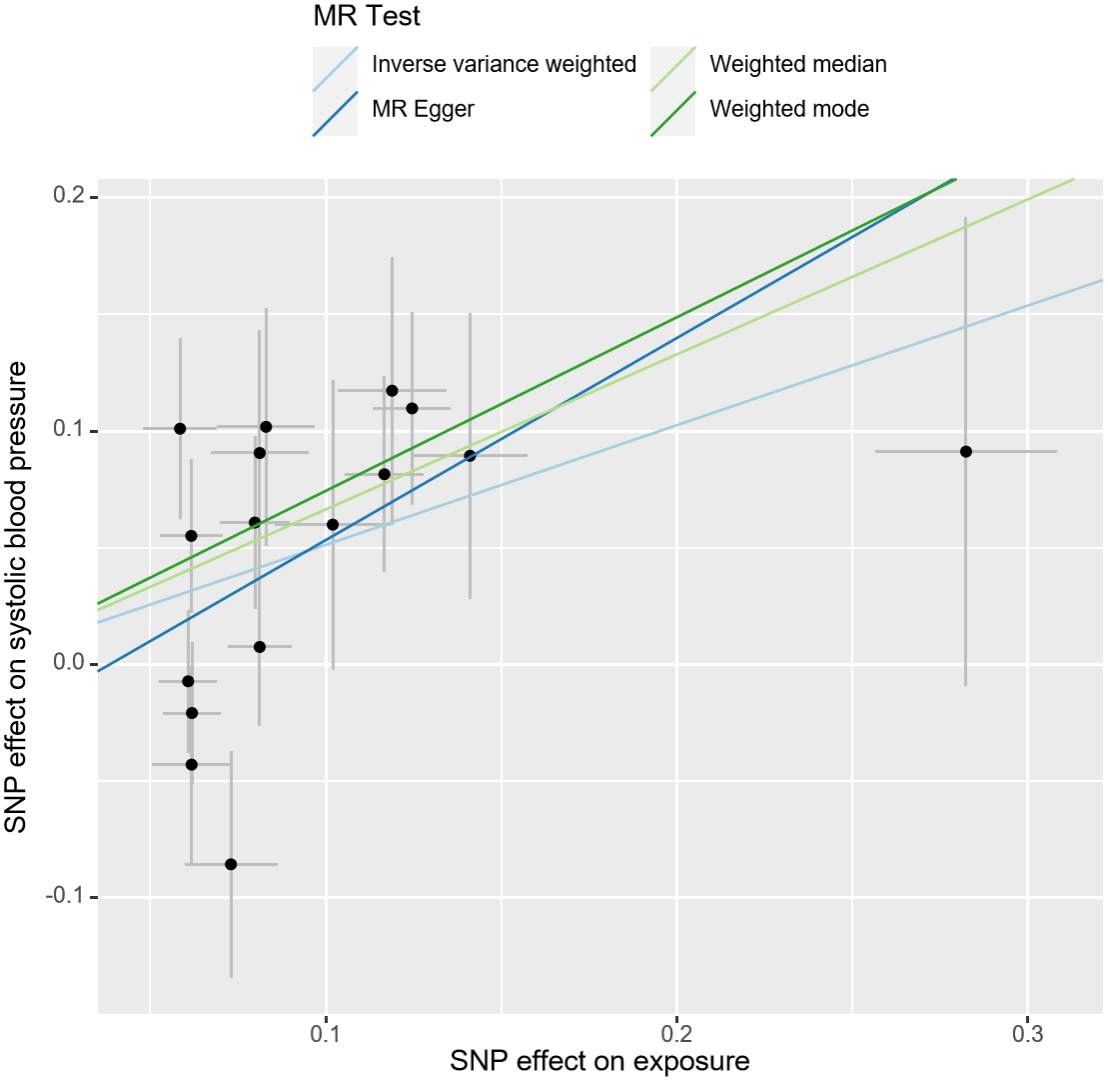
Scatter plot of the association of UL and SBP. The slope of the straight line indicates the magnitude of the causal association. UL, uterine leiomyoma; SBP, systolic blood pressure; SNP, single nucleotide polymorphisms; MR, Mendelian randomization.

**Figure 5:**
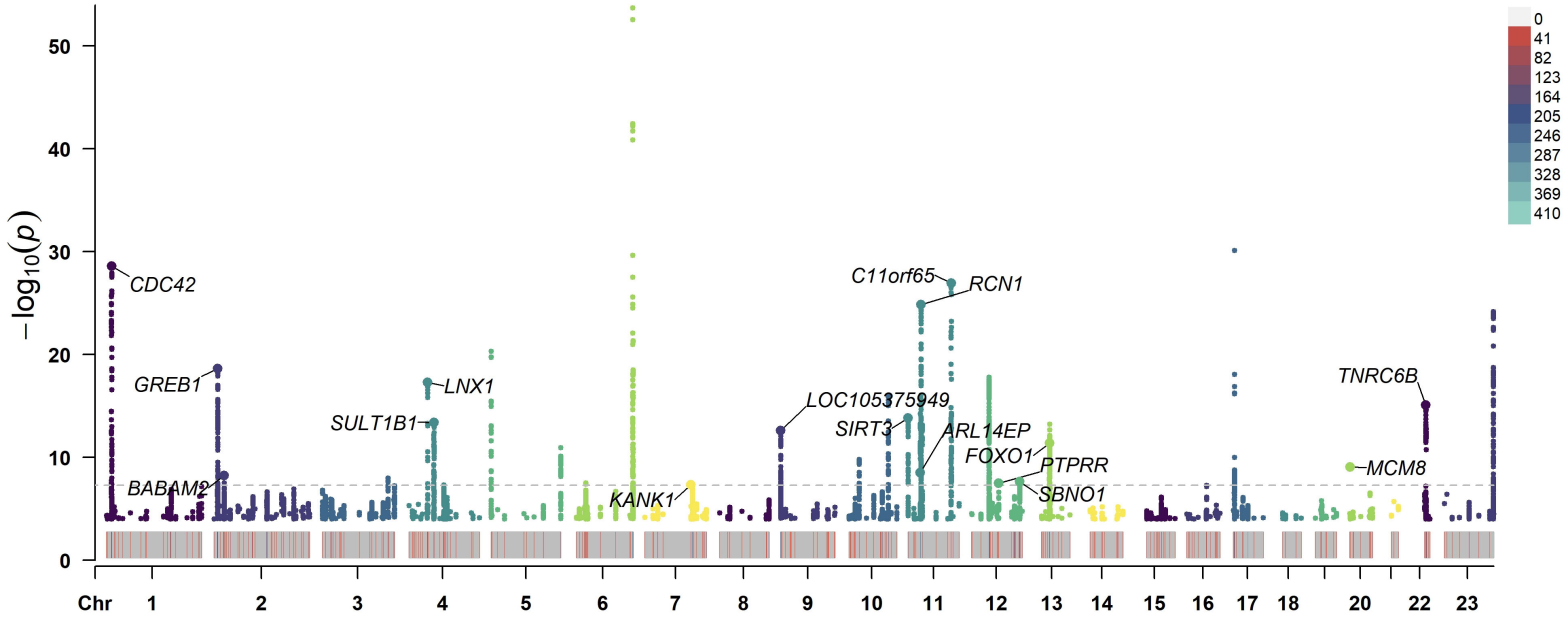
Manhattan plot of −log10 values using GWAS summary statistics of UL and SBP. The loci of 16 SNP that were significantly associated with UL and SBP is annotated in the plot. The horizontal axis represents the chromosome number. The dashed line indicates the *P*<5×10^−8^ threshold. GWAS, genome-wide association study; UL, uterine leiomyoma; SBP, systolic blood pressure; SNP, single-nucleotide polymorphism.

After the MR-PRESSO test, no outliers were found in the MR analysis of UL and the three subtypes of HDP. However, there existed outliers in the MR analysis of UL and hypertension (rs116251328, rs4325427, rs72709458), SBP (rs10508765, rs117245733, rs2131371, rs58415480, rs78378222), and DBP (rs117245733, rs35446936). After searching through the PhenoScanner V2 database for all instrumental variables, we identified and filtered out two SNPs (rs78378222, rs116251328), that had been found to be associated with BMI related phenotypes. No SNP had been found to be associated with phenotypes related to waist circumference. After removing these SNPs, the causal inference was re-conducted, and the main results are shown in **Figure 3**. The causal inference results of UL and hypertension and DBP were basically consistent with the results before removing the outliers. Interestingly, the IVW method of causal inference of UL and SBP suggested a positive casual relationship (OR =1.67, 95% CI: 1.24~2.25, *P*=0.0007). The MR-Egger method (OR=2.38, 95% CI: 1.03~5.46, *P*=0.06), weighted median method (OR=1.94, 95% CI: 1.40~2.70, *P*=8.34×10^-5^), and weighted model (OR=2.10, 95% CI: 1.33~3.33, *P*=0.006). All had consistent results with the IVW method. The Cochrane’s Q test showed *P*=0.06, and the *P*-value of the MR-Egger intercept test was 0.39, indicating that there was no heterogeneity or pleiotropy. The scatter plot and Manhattan plot are shown in **Figures 4** and **5**. It can be considered that there is a positive causal relationship between UL and SBP.

## Discussion

This study used large-scale GWAS data from public databases to explore the causal relationship between UL and each outcome (hypertension, HDP, SBP, and DBP) using two-sample MR methods. This analysis found that the occurrence of UL was positively associated with the risk of elevated SBP. No clear evidence of a causal relationship was found between UL and other outcomes.

UL is a common benign tumor in gynecology with a morbidity rate of up to 68.6% in certain area (8), while hypertension affects over 1 billion people worldwide (1). Both diseases have caused a significant medical burden globally. The relationship between UL and cardiovascular diseases has been studied for years (9–15), but the research conclusions remain controversial. Previous studies have reported that women with UL have higher SBP levels than women without UL (9, 10, 15), and similar results have been observed in studies of pregnant women (18). It is consistent with the results of our study, indicating that UL has certain effect on raising blood pressure. Another recent study has found that after surgical removal of UL, patients’ SBP decreased (41), further indicating a possible link between UL and SBP. This study did not find a statistical association between UL and hypertension, which is consistent with the results of Uimari et al. (14) and Laughlin-Tommaso et al. (15). This may be due to differences in blood pressure baselines among different populations in different samples, and the hypertensive effect of UL may not yet meet the diagnostic criteria for hypertension in different populations, leading to variance in study results.

The pathophysiological connection between UL and hypertension remains unclear, yet they share structural similarities. Uterine leiomyoma is typically a benign tumor caused by the growth of smooth muscle cells, while hypertension is also associated with abnormalities in vascular smooth muscle (42). Therefore, some scholars have proposed that the proliferation of uterine smooth muscle is similar to the changes of atherosclerotic (43). The study by Hoag et al. (44) has found that the expression level of creatine kinase (CK) in uterine leiomyoma tissue was higher than that in adjacent uterine muscle tissue, and CK could provide ATP for vascular smooth muscle contraction (45). Additionally, higher CK activity is associated with increased arterial contractility (46). It has also been found that angiotensin II receptors type 1 and type 2 are expressed in both the myometrium and uterine fibroids (47). It is well known that angiotensin II plays an important role in the development of hypertension, so the involvement of the renin-angiotensin-aldosterone system may also explain, to some extent, the pathophysiology of these two diseases. The angiotensin-converting enzyme inhibitors can inhibit the production of angiotensin II, which have been commonly used in the treatment of hypertension. A cohort study analyzed 353,917 participants has found that the use of angiotensin-converting enzyme inhibitors could reduce the risk of UL, which seems to further support the link between UL and hypertension (48). Besides, UL may also stimulate smooth muscle proliferation and vasoconstriction through various growth factors such as insulin-like growth factor-1 and platelet-derived growth factor (12). Further research is needed to explore the biological roles of the identified risk locipotential biological mechanisms connecting risk gene loci with UL and SBP.

The strength of this study is that it first analyzed the relationship between UL and blood pressure using the MR method. However, there are also some limitations. Firstly, despite various sensitivity analyses conducted, it should be noted that the existence of horizontal pleiotropy cannot be fully excluded, which may introduce bias into the results. Secondly, the genetic variants used in this study reflect the impact of UL on blood pressure, but further analysis of the impact of UL’s size, number, and location on blood pressure is lacking. Thirdly, the population of this study was derived from European ancestry. While this reduces population stratification bias, it may not be as reliable when extrapolated to other ethnic groups. Given that GWAS data for African populations are limited, and the sample size and case numbers of GWAS data for Asian populations are relatively small, future studies could pay more attention to expanding GWAS databases for Asians and Africans to further investigate the relationship between uterine leiomyomas and blood pressure in other populations. Besides, we hope that future researchers can conduct higher-quality and more detailed genome-wide association studies to identify genetic loci that affect the location, number, and size of uterine fibroids, so as to further explore the impact of different types of uterine fibroids on blood pressure.

The findings of this study provide robust causal evidence for the association between UL and blood pressure. This implies that UL is not merely a locally growing benign tumor, but it may also have certain impacts on systemic physiological indicators. Pulgar has indicated that UL might be a risk factor for the development of HDP (49). Consequently, in clinical practice, doctors need to pay closer attention to the blood pressure status of UL patients, particularly pregnant women combined with UL, and consider whether to reassess the patient’s treatment plan. For hypertensive patients who already diagnosed with UL, if there is a case of elevated blood pressure, after ruling out other potential causes for the increase, doctors should be on high alert and consider whether interventional treatment for UL is necessary to prevent further elevation of blood pressure.

However, it is essential to recognize that while Mendelian randomization studies can provide robust causal evidence, their results still need to be validated in larger-scale clinical trials. Therefore, future research should aim to further confirm this discovery and explore the underlying biological mechanisms, in order to provide more precise and effective strategies for the treatment of UL, hypertension, or HDP patients. More rigorous and comprehensive prospective large-scale longitudinal cohort studies can be conducted to clinically validate the relationship between UL and hypertension and HDP, and further analyze whether the size, number, and location of UL have different impacts on blood pressure. It can also be investigated whether there is a change in blood pressure after treatment for UL. This may reduce the incidence and prevalence of hypertension and HDP in patients with UL.

## Data availability statement

The datasets presented in this study can be found in online repositories. The names of the repository/repositories and accession number(s) can be found below: https://www.ebi.ac.uk/gwas/studies, GCST90038604, https://gwas.mrcieu.ac.uk/, ieu-b-38, https://gwas.mrcieu.ac.uk/, ieu-b-39.

## Ethics statement

Ethical approval was not required for studies on human participants, as only published GWAS summary statistics and ethically approved data obtained from FinnGen R9 biobank were used. These studies were conducted in accordance with local legislative and institutional requirements. The human samples used in this study were obtained from previous studies that had obtained ethical approval. No written informed consent from the subjects or their legal guardians was required for participation in this study.

## Author contributions

HX: Writing – review & editing, Visualization, Writing – original draft, Resources, Formal analysis, Data curation. YM: Resources, Writing – review & editing, Funding acquisition. YL: Writing – review & editing, Supervision, Project administration, Formal analysis. RL: Funding acquisition, Writing – review & editing. ZC: Methodology, Data curation, Conceptualization, Writing – review & editing. XX: Supervision, Writing – review & editing. XH: Supervision, Writing – review & editing, Funding acquisition. XW: Supervision, Project administration, Writing – review & editing, Funding acquisition.
